# Many-particle excitations in non-covalently doped single-walled carbon nanotubes

**DOI:** 10.1038/s41598-019-50333-7

**Published:** 2019-10-18

**Authors:** Timofei V. Eremin, Petr A. Obraztsov, Vladimir A. Velikanov, Tatiana V. Shubina, Elena D. Obraztsova

**Affiliations:** 1 Faculty of Physics of M.V. Lomonosov Moscow State University, Leninskie Gory str. 1, Moscow, 119991 Russia; 2 A.M. Prokhorov General Physics Institute of RAS, Vavilov str. 38, Moscow, 119991 Russia; 3 Moscow Institute of Physics and Technology, 9 Institutskiy per., Dolgoprudny, Moscow Region, 141701 Russia; 4 Department of Physics and Mathematics, University of Eastern Finland, Yliopistokatu 7, Joensuu, 80101 Finland; 5 National Research Nuclear University, Moscow Engineering Physics Institute, 31 Kashirskoe Highway, Moscow, 115409 Russia; 6 Ioffe Institute of RAS, Politechnicheskaya str. 26, St Petersburg, 194021 Russia

**Keywords:** Carbon nanotubes and fullerenes, Carbon nanotubes and fullerenes

## Abstract

Doping of single-walled carbon nanotubes leads to the formation of new energy levels which are able to participate in optical processes. Here, we investigate (6,5)-single walled carbon nanotubes doped in a solution of hydrochloric acid using optical absorption, photoluminescence, and pump-probe transient absorption techniques. We find that, beyond a certain level of doping, the optical spectra of such nanotubes exhibit the spectral features related to two doping-induced levels, which we assign to a localized exciton $X$ and a trion *T*, appearing in addition to an ordinary exciton ${E}_{1}$. We evaluate the formation and relaxation kinetics of respective states and demonstrate that the kinetics difference between *E*_1_ and *X* energy levels perfectly matches the kinetics of the state *T*. This original finding evidences the formation of trions through nonradiative relaxation via the $X$ level, rather than via a direct optical excitation from the ground energy state of nanotubes.

## Introduction

Multi-particle interactions exert a significant influence on physical properties of single-walled carbon nanotubes (SWNTs), as became clear after the first direct experimental confirmation of the excitonic nature of optical transitions in SWNTs^1^. Accounting for the electron-electron, electron-hole and exciton-phonon interactions allowed the successful explanation of a set of phenomena that were inexplicable with a simple non-interacting model. The ratio problem (the deviation of the second to first optical transition energy ratio from that predicted by commonly used theoretical models), a peculiarly low quantum yield of SWNT photoluminescence (PL) and the positions of phonon-side bands are among such phenomena^2,3^. Thus, many-body interactions in carbon nanotubes have become an increasingly active research area.

There is considerable interest in doped nanotubes, formed using various methods, such as chemical and gate doping, covalent functionalization, etc. Such modification methods may strongly impact the energy structure of SWNTs and yield new complex quasiparticles. Several different interpretations of these doping-induced many-particle energy levels can be found in the literature^4–20^.

Theoretical studies by Rønnow *et al*. showed that the interaction of an exciton with either an electron or a hole in a SWNT may lead to the formation of a negatively or positively charged quasiparticle (known as a trion), respectively, detectable even at room temperature^4^. The first experimental confirmation of this prediction was done by Matsunaga *et al*., who observed new peaks in absorption and PL spectra of SWNTs after p-doping with 2,3,5,6-tetrafluoro-7,7,8,8-tetracyanoquinodimethane (F4TCNQ) and hydrochloric acid (HCl)^5^. These new peaks were assigned to the optical transitions between the ground (non-excited) energy state of the nanotubes and a new doping-induced energy state, located approximately 100–200 meV (depending on the nanotube diameter) below the bright exciton, and attributed to the positive trion. It was also reported later that trions can be generated by all-optical excitations^6,7^.

Later, applying doping methods such as gate-doping, electrochemical doping, chemical doping with F4TCNQ and HCl, several groups reported new features in linear absorption^8–10^ and PL spectra^11–14^ and attributed them to a direct optical excitation and a radiative decay of trions, respectively. Compared to the main exciton peak, such new peaks exhibit increased intensity with increased doping concentration, while the total absorbance and luminescence signal decreases.

On the other hand, a set of works have shown that the covalent functionalization of SWNTs leads to the appearance of very similar spectral features, i.e. the satellite peaks located 100–200 meV below the main exciton peak^15–20^. In these works, such peaks are attributed not to the trions but to the excitons, localized on defects which are introduced into the SWNT wall structure by covalent functionalization. The lower energy position of such excitons is usually explained as a result of modified potential in the defect vicinity. The exact position of these defect-localized excitons can be sensitive to the type of bonded functional group^18,20^.

It is important to note that localization of excitons is not limited to the vicinity of defects induced by a covalent functionalization. A theoretical investigation by Tayo at al. indicated that charged particles, physisorbed on the surface of SWNTs, might play the role of localization centres for excitons^21^. It was also found experimentally in electrochemically doped nanotubes that both excitons and trions are spatially confined because of adsorbed ions, even without chemical bonding with the nanotube wall^22^. The temperature dependence of new doping-induced peaks in PL spectra confirmed the spatial localization of corresponding quasiparticles^23^.

Brozena *et al*. observed defect-localized excitons in diazonium-functionalized SWNTs and the appearance of a second extra PL peak after chemical doping (reducing) of diazonium-functionalized SWNTs. This second peak was attributed to trions^24^. Thus, both localized excitons and trions can coexist in nanotubes modified with such a two-step method. A combination of covalent aryl functionalization and gate doping leads to the existence of both localized excitons and trions, as was reported by Shiraishi *et al*.^25^. Very recently, two different doping-induced levels were also reported by Bai at el. for SWNTs homogeneously non-covalently doped using K_2_IrCl_6_^26^; however, these levels were attributed to hole-polaron-dressed excitons and trions.

Bai *et al*. reported a one-picosecond trion formation time after the excitation of either an ordinary exciton or a hole-polaron-dressed exciton and concluded that trions are not formed as a result of the direct optical transition from the ground energy level, but rather via the intermediation of the hole-polaron-dressed exciton state. This finding contradicted previous works, since Nishihara *et al*. reported that trions can be generated via direct optical transitions from the ground energy level, using a model accounting for the dark exciton level^9^, and Koyama *et al*. revealed that the trion level is occupied almost immediately (delay is less than 100 fs) after the occupation of the main exciton level^8^.

In this work, we report the first observation of two doping-induced levels of different natures in SWNTs non-covalently doped with HCl. We ascribe these levels to the localized excitons and trions. We compare the ultrafast formation and relaxation dynamics of these photo-excitations in (6,5)-SWNTs doped by HCl and reveal that the occupation of the trion energy level occurs with a delay of 1 ps via the intermediary *X* level. Together with absorption and PL data, this finding indicates that the trions do not form via the direct optical excitation from the ground energy state in SWNTs noncovalently doped by HCl.

## Results and Discussion

An aqueous suspension of (6,5)-enriched SWNTs coated with sodium dodecyl sulfate (SDS) was used in this research. Doping of the nanotubes was achieved by adding HCl to this suspension.

**Figure 1:**
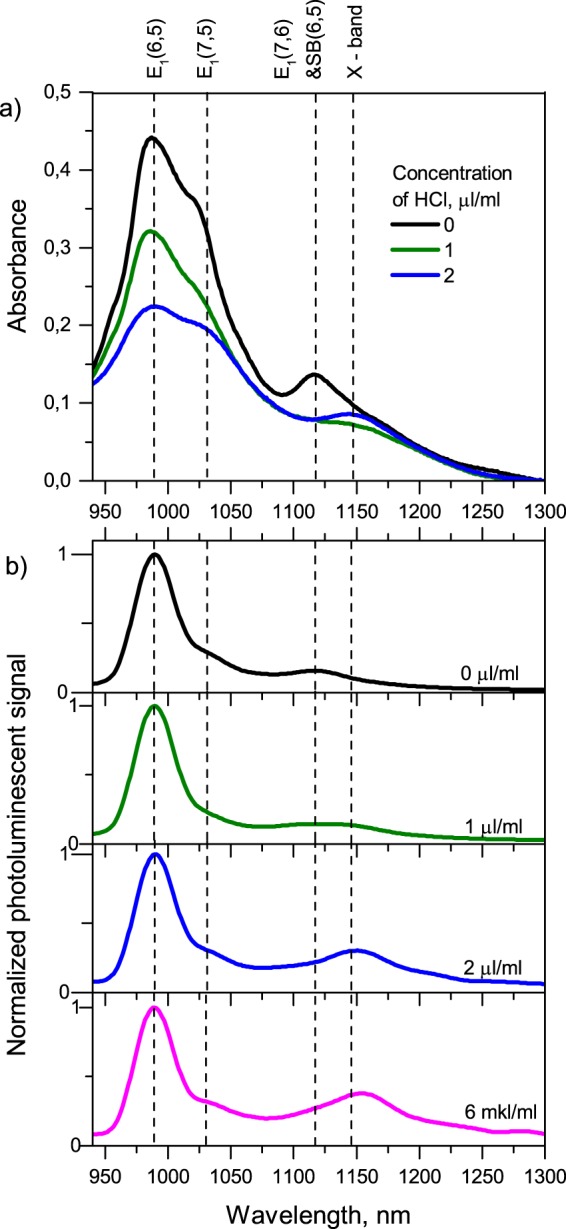
(**a**) Optical absorption spectra and (**b**) normalized PL spectra of the SWNT suspensions with different concentration of HCl.

Figure 1a shows the optical absorption spectra of SWNT suspensions with different concentrations of HCl. The three peaks labelled *E*_1_(6,5), *E*_1_(7,5) and *E*_1_(7,6) in the spectrum of a non-doped sample (black line) correspond to the excitation of the first bright excitonic states in nanotubes with the indicated chiral indexes. With increasing concentration of HCl, one can observe a gradual suppression of all three excitonic peaks. At a concentration of 1 µl/ml, the E_1_(7,6) peak is totally suppressed (dark green line). At a higher concentration (2 µl/ml), the gradual rise of a new peak, labelled X, located around 1140–1160 nm is observed (blue line).

Figure 1b shows the normalized PL spectra of suspensions with different concentrations of HCl under the resonant excitation of the second bright exciton E_2_ in (6,5) nanotubes (570 nm). The brightest peak at 990 nm corresponds to the radiative decay of the first bright exciton in (6,5)-SWNTs. A faint peak around 1025–1030 nm should be assigned to the emission from (7,5) nanotubes, which appeared due to an exciton energy transfer (EET) from a (6,5) nanotube^27^. A spectral feature at 1120 nm, labelled E_1_(7,6) + SB(6,5), might consist of two overlapped peaks: a signal of an EET process from (6,5) to (7,6) nanotubes and a phonon-side photoluminescence band^28,29^, associated with (6,5) nanotubes.

In the rest of this paper, we omit chiral indices (n,m) in E_1_(n,m), as we only discuss (6,5) nanotubes. When the HCl concentration is greater than 1 µl/ml, we observe the gradual rise of a new PL peak around 1140–1160 nm, labelled *X*. We suggest that the observable red-shift of the *X* band from 1140 nm to 1160 nm is due to the influence of HCl on the local dielectric constant and, consequently, to the screening efficiency^30^.

**Figure 2:**
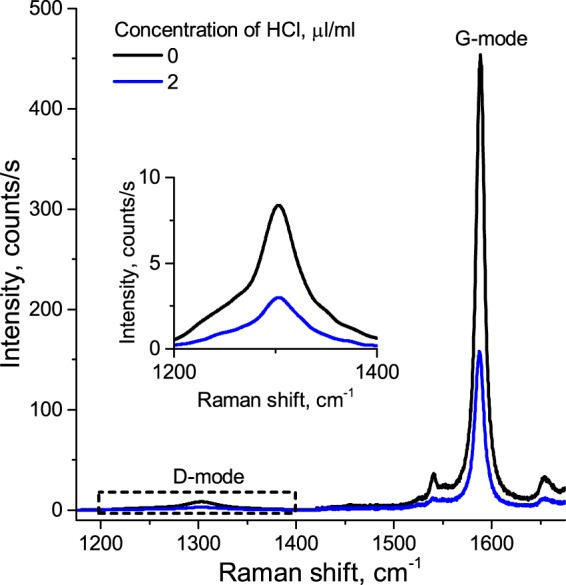
Raman spectra of the initial SWNT suspension (black line) and SWNT suspension with HCl (blue line). Inset: zoomed D-mode spectral region. The excitation wavelength is 633 nm.

In order to check the influence of HCl on the structure of SWNT, we performed Raman measurements (see Fig. 2.). Since we do not observe a rise of the “defective” D-mode compared to the G-mode with increasing of HCl concentration, we conclude that adding of HCl does not lead to the formation of defects in the structure of SWNT.

**Figure 3:**
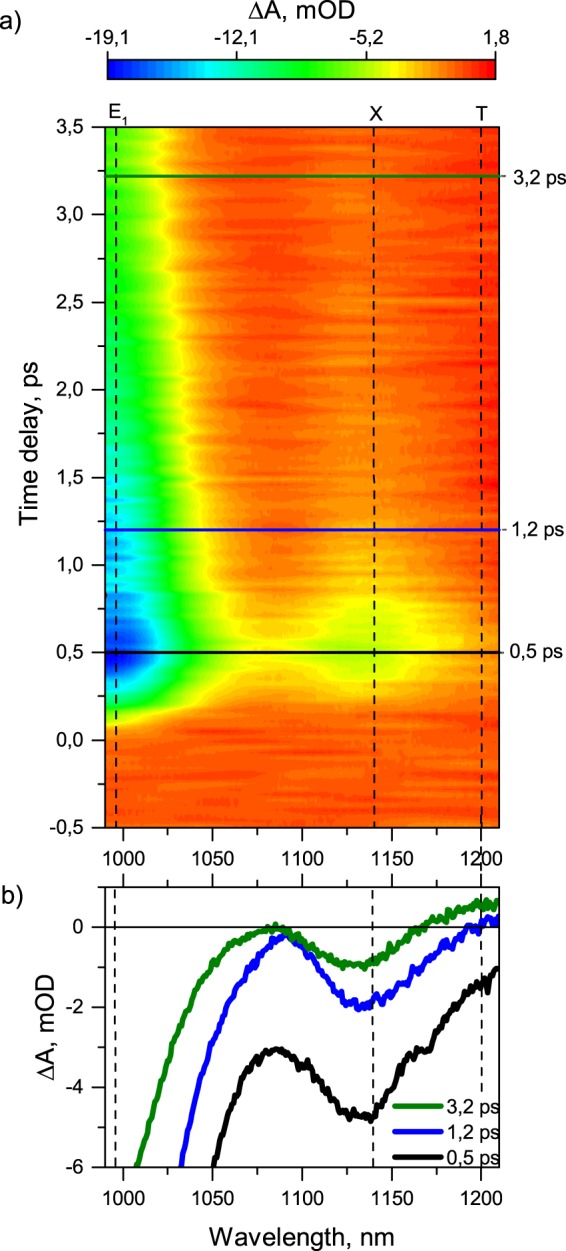
(**a**) Time dependence of the absorbance change signal of the suspension pumped at 570 nm. (**b**) Differential absorption spectra of the suspension taken at several different delay times.

The suspension with 2 µl/ml HCl exhibiting the bright *X* spectral features (solid blue lines in Fig. 1a,b) was investigated by the pump-probe technique. The colour map in Fig. 3a shows a dependence of the induced sample optical density on the time delay between a pump pulse centred at E_2_ resonance of (6,5) nanotubes (570 nm) and a probe pulse covering the spectral region 990–1210 nm. The occupancy of the first bright excitonic level of (6,5) nanotubes reveals itself as an induced transmittance at 990 nm. Since both optical absorption and PL data demonstrate that, under concentrations of HCL higher than 1 µl/ml, the (6,5)-SWNTs exhibit an additional optically active energy level *X* appearing as a peak around 1140–1160 nm, we assign an induced transmittance signal centred approximately at 1140 nm in Fig. 3a to the same energy level X.

The differential absorption spectra taken at different delay times between pump and probe pulses (horizontal cuts in Fig. 3a) are shown in Fig. 3b. One can see that along with the induced transmittance features *E*_1_ and *X* there is a significant induced absorption signal around 1200 nm, labelled *T*.

**Figure 4:**
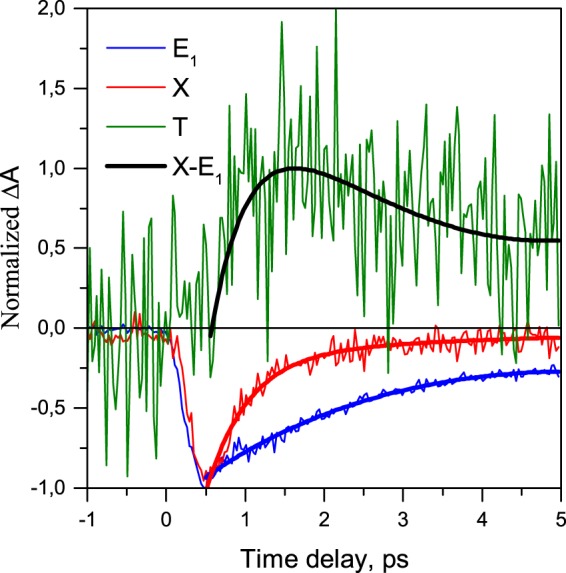
Measured dynamics of E (blue lines), X (red lines) and T energy level (green line). The black line shows the difference between X and E_1_ dynamics.

The time evolution of *E*_1_ and *X* energy levels are obtained as vertical cuts in Fig. 3a at 990 nm and 1140 nm and plotted as blue and red lines in Fig. 4, respectively. Thin noisy lines are experimental data, while the solid lines refer to bi-exponential fitting curves.

Although the *T* spectral feature is clearly observable at delay times exceeding 1 picosecond (green line in Fig. 2b), at shorter time delays it is buried under the strong induced transmittance signal *X* due to, inter alia, a lack of material homogeneity. Therefore, the time evolution of the *T*-associated energy level cannot be obtained simply from a vertical cut of Fig. 3a at 1200 nm, and the side slope signal of the *X* band should be subtracted with the necessary normalization.

**Figure 5:**
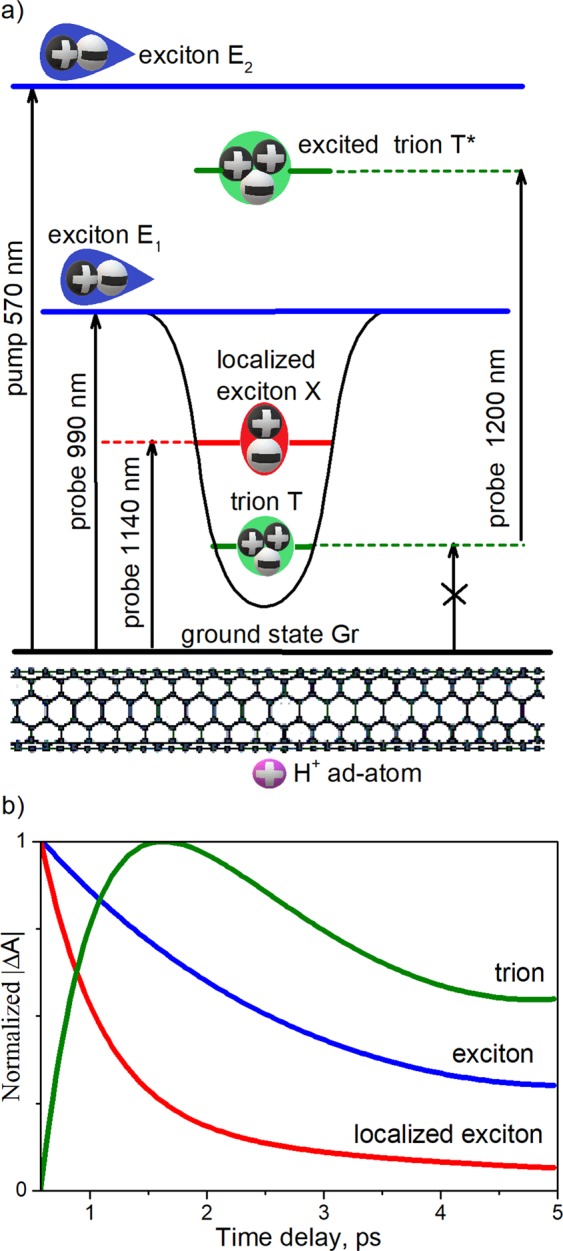
(**a**) Proposed simplified scheme of energy structure of SWNTs doped with HCl. (**b**) Established dynamics of main energy levels in the system (**b**).

Applying such a procedure, we obtain dynamics of the *T*-associated energy level (as shown by green line in Fig. 4). It is clearly seen that the formation of *T*-states occurs with a delay of about 1 ps after the formation of the ordinary *E*_1_ exciton and *X* states. This finding signifies that the *T* spectral feature does not correspond to either *X* or *E*_*1*_ energy levels, but is associated with another energy level (see Fig. 5).

Similar dynamics of the ordinary exciton and the doping-induced states was recently observed by Bai *et al*.^26^ in highly electrically and geometrically homogeneous (6,5)-SWNTs non-covalently doped with K_2_IrCl_6_. Thus, here we confirm their findings for the case of less homogeneous (6,5)-SWNTs, doped with HCl.

The black line in Fig. 4 shows the difference in occupation of *E*_1_ and *X* energy levels and indicates faster relaxation dynamics of the *X* energy level. The difference in relaxation dynamics of these two levels (black line in Fig. 4) precisely matches the dynamics of the *T* energy level (green line in Fig. 4). This original finding strongly supports the appearance of additional ultrafast energy relaxation channel of *X* states through the doping-induced energetically lower lying states, namely, the formation of the *T* states.

The absence of a distinct signal at 1200 nm in PL and linear optical absorption spectra indicates that optical transitions between the ground energy level (*Gr*) of the nanotubes and the T energy level (*Gr* = > *T* and *T* = > *Gr* transitions) have very low oscillator strength at 1200 nm compared to the oscillator strength of *Gr* = > *X* and *X* = > *Gr* transitions at 1140 nm. Thus, in our experimental conditions, the *T* states are formed via a nonradiative relaxation from the *X* energy level, but not via the direct optical transition from the *ground* energy level of nanotubes (see crossed-out arrow in Fig. 5).

We further speculate on the physical nature of *X* and *T* energy levels (see Fig. 5) and compare our results with those obtained in previous works. The authors who attributed the *X* energy level to trion^5,8–14^, did not observed a second doping-induced level *T*, as we do in this work. In contrast, in works where two different doping-induced levels were observed^24–26^, and also in several other works^15–20^, the *X* energy level is ascribed either to a defect-localized exciton or to a hole-polaron-dressed exciton. Since we do not observe a rise of the “defective” D-mode in the Raman spectra with HCl added (see Fig. 2), we reject the hypothesis of defect-localized excitonic states^12^. However, both theoretical^21^ and experimental^22^ studies have shown that excitons may localize due to the impact of ions adsorbed on the surface of SWNTs, even without covalent bonds. Thus, one should ascribe the *X* energy level either to the hole-polaron-dressed exciton or to the excitons localized on the sites of physically adsorbed H^+^ ions. Due to the theoretical support, we are inclined to favour the latter hypothesis, although further experimental studies, such as Fourier-transform infrared spectroscopy and x-ray photoelectron spectroscopy measurement, should be done to clearly specify the physical nature of the *X* energy level.

Regarding interpretation of the second doping-induced level *T*, located energetically below the X level, we follow previous works and ascribe it to the trion energy level^24–26^. However, in contradiction to some other works^24,25^, but in consonance with Bai *et al*.^26^, we find that the *Gr* = > *T* and *T* = > *Gr* transitions are optically inactive. This discrepancy might be caused by specific doping techniques and environmental circumstances. Following Bai *et al*.^26^, we attribute the *T* transient absorption spectral feature around 1200 nm to the *T* = > T* optical transition, where T* denote an excited trion state (see Fig. 5). Such an interpretation is in agreement with theoretical work^31^, claiming that not only one but rather a set of trion levels exist in SWNTs.

## Conclusion

To summarize, we provide new physical insight on the energy structure of SWNTs doped with hydrochloric acid and on the physical nature and behaviour of the corresponding many-particle excitations in such modified nanomaterial. We observed two doping-induced energy levels *X* and *T* in HCl-doped (6,5)-SWNTs, which we cautiously ascribe to the exciton, localized on the physisorbed H + ion, and to the trion, respectively. We present an original finding showing that the dynamics of the trion states matches the difference between the dynamics of the ordinary exciton *E*_1_ and the exciton *X*, localized on the physisorbed ion. Thus, our results strongly suggest that the SWNT trion energy level *T* in HCl-doped (6,5)-SWNTs is occupied via relaxation from the *X* energy level, but not via a direct optical excitation from the ground energy level of nanotube. These findings significantly contribute to understanding the energy structure of doped SWNTs and many-body interactions in low-dimensional materials, although further studies should be aimed at the accurate attribution of energy levels in SWNTs, doped by different methods.

## Materials and Methods

A powder of (6,5)-enriched CoMoCat single-walled carbon nanotubes (Sigma-Aldrich) was dissolved in an aqueous 2% solution of SDS in concentration of 0.05 µg/ml involving 4 hours of tip sonication. The obtained suspension was ultracentrifuged (120000 g) for 1 hour, and supernatant was collected for further research. Doping was performed by adding concentrated hydrochloric acid into a quartz cuvette containing 1 ml of the suspension. The linear optical absorption and PL measurements dealt with the quartz cuvette, while for the pump-probe measurements the suspension was placed between two glass windows, making the optical length of the sample of 2 mm.

The linear optical absorption measurements were done using a two-channel PerkinElmer Lambda 950 spectrophotometer. For PL measurements a “Nanolog-4” spectrofluorimeter (*Horiba*) was used, with a xenon lamp as the excitation light source and a nitrogen-cooled InGaAs matrix as the detector. The installation has already been used in our previous studies^32,33^.

To perform the time-resolved measurements and to reveal the ultrafast dynamics of different relaxation channels in initial and HCl doped SWNT we employed a multicolor transient absorption pump−probe setup based on Ti:sapphire 40 fs laser. A detailed description of the method and setup we reported previously and can be found elsewhere^34,35^. The femtosecond pulses with a central wavelength tunable in the 1000–1700 nm range and a femtosecond continuum were employed as pump and probe, respectively. The pump pulses were delivered by an optical parametric amplifier (OPA), which was excited by a Ti:sapphire regenerative femtosecond amplifier (800 nm wavelength, 40 fs pulse duration, 1 kHz repetition rate). The probe continuum pulses were generated by focusing the beam (at the wavelength of 800 nm and with an average power of 100 mW) in a sapphire crystal. The diameter of the pump beam at the sample surface was about 500 μm. The femtosecond continuum was used to probe the absorbance change (ΔA) in a wide spectral range spanning from 900 nm up to 1300 nm. The visible part of the continuum generated in the sapphire crystal was removed from the probe channel using a long-pass filter. The pump and probe beams were polarized collinearly. The time delay between the probe and the pump pulses was controlled by a rapid motorized delay line. The pump-induced change of the probe absorption was detected with an IR-spectrometer (CDP ExciPro 2012). All measurements were performed at room temperature.

## Data Availability

The datasets generated and analysed during the current study are available in the Google Drive repository via the following link: https://drive.google.com/file/d/1C-OrsueR7Y_cxvAkLPVPYJEnJ1p_TVUm/view?usp=sharin.
